# Prevalence and sociodemographic correlates of physical activity and sitting time among South American adolescents: a harmonized analysis of nationally representative cross-sectional surveys

**DOI:** 10.1186/s12966-022-01291-3

**Published:** 2022-05-08

**Authors:** Raphael H. O. Araujo, André O. Werneck, Luciana L. Barboza, Robinson Ramírez-Vélez, Clarice M. L. Martins, Rafael M. Tassitano, Ellen C. M. Silva, Gilmar M. de Jesus, Thiago S. Matias, Luiz R. A. de Lima, Javier Brazo-Sayavera, Danilo R. Silva

**Affiliations:** 1 Graduation Program in Health Sciences, Londrina State University, Londrina, Brazil; 2 Center for Epidemiological Research in Nutrition and Health, Department of Nutrition, School of Public Health, University of São Paulo (USP), São Paulo, Brazil; 3 Postgraduate Program in Physical Education, University of Brasília (UnB), Brasília, Brazil; 4 Navarrabiomed, Hospital Universitario de Navarra (HUN), Navarra Institute for Health Research (IdiSNA), Universidad Pública de Navarra (UPNA), Pamplona, Navarra Spain; 5 Facultad de Ciencias de la Educación, Unidad Central del Valle del Cauca (UCEVA), Tuluá, Valle del Cauca, Colombia; 6 Department of Physical Education, Federal University of Paraíba, Brazil, and Research Center in Physical Activity, Health and Leisure, Faculty of Sports, University of Porto, Porto, Portugal; 7 Department of Physical Education, Federal Rural University of Pernambuco, Recife, Brazil; 8 Department of Health, State University of Feira de Santana, Feira de Santana, Brazil; 9 Department of Physical Education, School of Sports, Federal University of Santa Catarina, Florianópolis, Brazil; 10 Institute of Physical Education and Sport, Federal University of Alagoas, Maceió, Brazil; 11 PDU EFISAL, Centro Universitario Regional Noreste, Universidad de la República, Rivera, Uruguay and Department of Sports and Computer Science, Universidad Pablo de Olavide (UPO), Seville, Spain; 12 Department of Physical Education, Federal University of Sergipe, São Cristóvão, Brazil

**Keywords:** Adolescence, Physical activity, Transportation, Sedentary behavior, South America

## Abstract

**Background:**

To identify the prevalence and sociodemographic correlates of different domains of physical activity (PA) and higher sitting time among South American adolescents.

**Methods:**

Data from national surveys of 11 South American countries were analyzed, and comprised information on 166,901 adolescents. PA (≥ 60 min/day of moderate-vigorous PA), physical education classes (PEC) (≥ 3 classes/wk), active commuting to school (≥ 1 d/wk), and higher sitting time (≥ 3 h/d) were self-reported. Sociodemographic correlates, such as gender, age, and food security status were explored using a random effect meta-analysis for logistic parameters.

**Results:**

Recommended PA ranged between 7.5% (Brazil) and 19.0% (Suriname). Peru (2.2%) and Guyana (43.1%) presented the lowest prevalence of PEC and active commuting to school, respectively. Higher sitting time was less prevalent in Bolivia (24.6%) and more prevalent in Argentina (55.6%). Compared to girls, boys were more prone to reach recommendations for PA [OR = 1.94(1.65;2.28)]; to reach ≥ 3 PEC [OR = 1.17(1.04;1.33)] and to be active in commuting to school [(OR = 1.14(1.06;1.23)], but less prone to higher sitting time [OR = 0.89(0.82;0.96)]. Older adolescents had less odds of reach PA guidelines [OR = 0.86(0.77; 0.97)] and accumulated higher sitting time [OR = 1.27(1.14;1.41)]. Adolescents with food insecurity reported more PEC [OR = 1.12(1.04;1.21)] and active commuting to school [OR = 1.12(1.02;1.22)] but had less higher sitting time than their food security pairs [OR = 0.89(0.81;0.98)].

**Conclusions:**

Few adolescents reach the PA recommendation. Actions aiming the promotion of PA and the reduction of sitting time must consider girls and older adolescents as target groups, as well as the specifics of each country.

**Supplementary Information:**

The online version contains supplementary material available at 10.1186/s12966-022-01291-3.

## Background

Insufficient physical activity (PA) and higher sitting time are movement behaviors associated with several negative health outcomes during adolescence, such as impairment in cardiometabolic parameters [1, 2], and mental [3] and cognitive health [4]. The World Health Organization recommends that adolescents should accumulate on average 60 min per day of moderate-vigorous PA, as well as limit time spent on sedentary behaviors, especially recreational screen time [5]. Although the health benefits and recommendations on movement behaviors are well recognized, the promotion of an active lifestyle among adolescents is still a challenge worldwide [6].

In this sense, understanding the barriers and facilitators of PA in adolescents is an important step in the effective implementation of guidelines in different contexts, given that multiple factors can influence movement behaviors. Studies have been investigating the correlates and determinants of movement behaviors in different domains, indicating differences between countries [7–10]. For example, there is gender inequality, with girls presenting lower PA in different domains and higher sitting time in most countries; however, some countries and regions present higher gender inequality related to PA than others [9]. These differences directly affect interventions and public health policies, showing that there is not a “one-size-fits-all” way to promote active living, and that target groups for intervention strategies should be specifically guided by these correlates.

Several studies on the correlates of PA and sedentary behavior among adolescents have been conducted in developed countries in Western Europe and North America, while less affluent regions of the world have been underrepresented [7, 11]. South America, for example, is a sub-continent that covers 12% of the world population, characterized by increased social inequality [12] and a recent and accelerated urbanization process, leading to this area being the most urbanized region in the world [13]. Beyond the general inequalities, there are also marked regional, cultural, and economic differences between the countries [14], which can potentially change the association between the sociodemographic correlates and movement behaviors [15].

Previous cross-national studies involving South American countries found gender-inequalities, with boys practicing higher PA [8, 10, 16], and girls presenting higher sitting time than boys [8, 10]. Concerning economic inequalities, wealthier adolescents (boys and girls) presented higher PA outside school than their less wealthy peers [16]. However, there are still flaws in the previous studies that warrant further investigation. First, these studies had a more general focus (e.g., global and Latin America levels) and the associations between the sociodemographic correlates and movement behaviors in South America are still little explored [17]. Second, the association between age and movement behaviors was not investigated with harmonized datasets.

To support research on movement behaviors in South America, the South American Physical Activity and Sedentary Behavior Network (SAPASEN) [18] was established in 2018, seeking to use nationally representative datasets to provide continuous evidence. Thus, the current study aims to identify the prevalence and sociodemographic correlates of domain-specific PA and sitting time among South American adolescents.

## Methods

### Sample

This study performed a pooled analysis of cross-sectional data of national surveys from 11 South American countries (Argentina, Bolivia, Brazil, Chile, Colombia, Ecuador, Guyana, Paraguay, Peru, Suriname, and Uruguay). Data from Argentina (2018), Bolivia (2012), Chile (2013), Guyana (2010), Paraguay (2017), Peru (2010), Suriname (2016), and Uruguay (2012) were obtained from the Global School-based Student Health Survey (GSHS) [19]. Data from Brazil were from the 2015 Brazil National School Health Survey (*Pesquisa Nacional de Saúde do Escolar – PeNSE)* [20]. Data from Colombia were from the 2017 Colombia National School Health Survey (*Encuesta Nacional de Salud Escolar* – ENSE) [21]. Data from Ecuador were from the 2018 Ecuador National Health and Nutrition Survey (*Encuesta Nacional de Salud y Nutrición*—ENSANUT) [22]. All surveys were approved by ethics committees in their respective countries. Details about their sampling processes are presented in Supplementary Material A.

To harmonize the datasets, adolescents ≤ 11y and those with no data (including exposures and outcomes) were excluded. The final sample included 166,901 adolescents from Argentina (*n* = 52,423), Bolivia (*n* = 3,241), Brazil (*n* = 14,321), Chile (*n* = 1,878), Colombia (*n* = 72,808), Ecuador (*n* = 8,999), Guyana (*n* = 2,193), Paraguay (*n* = 2,848), Peru (*n* = 2,793), Suriname (*n* = 2,004), and Uruguay (*n* = 3,393). More details on missing values are presented in Supplementary chart 1 and sociodemographic characteristics of the countries are presented in Supplementary chart 2.

### Physical activity

PA was assessed by the question: “*During the past 7 days, on how many days were you physically active for a total of at least 60 min per day?*”; those who met at least 60 min/day in the last seven days were classified as “active” [8]. The ENSANUT survey did not consider the time spent in physical education classes in the total PA indicator. In this sense, the question used by ENSANUT was “*During the past 7 days, on how many days were you physically active for a total of at least 60 min per day? (exclude physical education classes at school)*”.

Active commuting to school was defined by the question: “*During the past 7 days, on how many days did you walk or ride a bicycle to and from school?*”; those who went to/from school ≥ 1 time per week were classified as “active commuting”[23].

Participation in physical education classes, in GSHS and ENSE surveys, was defined by the question: “*During this school year, on how many days did you go to physical education classes each week?*”. In the PeNSE survey, the question used was: “*During the last seven days, on how many days did you go to a physical education class at school?*”. The ENSANUT used two questions: a) “*in a normal week, when you go to school, do you go to the physical education classes?*”; b) “*on how many days did you go to a physical education class per week?*”. Those who did not go to the physical education class in a normal week (~ 3%) were classified as 0 days. For all datasets, we used the cut-off of three or more classes per week [8].

### Sitting time

Sitting time was defined by the question: “*How much time do you spend during a typical or usual day sitting and watching television, playing computer games, talking with friends, or doing other sitting activities?*”. The GSHS did not consider sitting time in school and doing the homework. The PeNSE did not consider Saturday, Sunday, holidays, and sitting time in school. We used the cut-off of three or more sitting hours per day [8].

### Sociodemographic correlates

The socio-demographic characteristics of gender and age group (12–13; 14–15; 16 +) were collected. The question about food insecurity was used as a proxy of socioeconomic status: “*During the past 30 days, how often did you go hungry because there was not enough food in your home?”;* this variable was coded as “never/rarely” (with food security) and “sometimes/most of the time/always” (with food insecurity) [24].

### Statistics

Descriptive statistics were performed using absolute and relative frequencies and their respective 95% confidence intervals. To identify the association between sociodemographic correlates with domain-specific PA and sedentary behavior, logistic regression models were used, reporting odds ratios. We conducted a random effect meta-analysis for logistic parameters using the command “metan”. All analyses included sample weight for each country and were conducted using STATA 15.0.

## Results

Table 1 presents the characteristics of the sample. The prevalence of active adolescents ranged from 7.5% (CI95% 6.9; 8.1, Brazil) to 19.0% (CI95% 16.6; 21.6, Suriname). The lowest prevalence of three or more physical education classes per week was observed in Peru (2.2%, CI95% 1.0; 4.9), and the highest in Argentina (37.5%, CI95%, 35.9; 39.1) and Colombia (37.5%, CI95%, 36.9; 39.0). Guyana (43.1%, CI95% 36.8; 49.7) reported the lowest percentage of active commuting to school. Regarding three or more hours per day of sitting time, Bolivia (24.6%, CI95% 21.9; 27.5) and Peru (28.8%, CI95% 25.6; 32.3) presented the lowest prevalence, while Argentina (55.6%, CI95% 53.8; 57.3) and Chile (54.1%, CI95% 50.4; 57.7) presented the highest.

**Table 1:** Sample characteristics according to gender, age group, food insecurity, physical activity, physical education, active commuting to school, and sitting time (*n* = 166,901)

	Gender	Age group			Food insecurity	Physical activity	Physical education	Active commuting to school	Sitting time
	Girls	12–13 y	14-15y	≥ 16y	Yes	Active	≥ 3 classes/week	≥ 1 travel/week	≥ 3 h/day
Argentina	47.6 (46.2; 49.1)	20.7 (19.1; 22.5)	46.9 (45.8; 48.0)	32.3 (30.6; 34.1)	11.3 (10.4; 12.4)	16.2 (15.5; 17.0)	37.5 (35.9; 39.1)	67.8 (65.3; 70.2)	55.6 (53.8; 57.3)
Bolivia	48.9 (46.7; 51.2)	22.1 (16.1; 29.6)	57.3 (52.0; 62.4)	20.6 (16.0; 26.1)	26.6 (24.5; 29.0)	14.1 (12.4; 16.0)	31.4 (29.3; 33.6)	64.63(60.5; 68.5)	24.6 (21.9; 27.5)
Brazil	49.0 (47.9; 50.2)	27.4 (26.6; 28.7)	31.2 (30.2; 32.3)	41.3 (40.2; 42.5)	12.0 (11.3; 12.9)	7.5 (6.9; 8.1)	9.6 (9.0; 10.2)	58.0 (57.0; 60.0)	50.6 (49.4; 51.7)
Chile	48.23(41.1; 55.4)	22.0 (15.9; 29.9)	34.6 (27.7; 42.2)	43.4 (36.4; 50.7)	8.1 (5.5; 10.1)	13.7 (11.9; 15.7)	32.93(30.6;35.3)	62.4 (32.6; 42.9)	54.1 (50.4; 57.7)
Colombia	53.6 (53.0; 54.2)	20.7 (20.1; 21.1)	43.5 (42.9; 44.1)	35.9 (35.4; 36.5)	7.3 (7.0; 7.7)	15.0 (14.6; 15.5)	37.5 (36.9; 39.0)	62.6 (62.1; 63.2)	46.5 (45.9; 47.1)
Ecuador	48.2 (46.5; 50.0)	35.7 (34.0; 37.4)	34.2 (32.5; 35.9)	30.1 (28.5; 31.7)	-	9.7 (8.6; 10.9)*	27.1 (25.5; 28.7)	50.2 (48.4; 52.0)	35.5 (33.8; 37.2)
Guyana	51.8 (48.9; 54.8)	24.2 (19.9; 29.1)	60.3 (55.7; 64.8)	15.5 (11.4; 20.7)	33.0 (26.9; 39.8)	15.8 (12.8; 19.2)	18.9 (16.1; 22.1)	43.1 (36.8; 49.7)	36.0 (31.5; 40.8)
Paraguay	51.7 (49.4; 54.0)	20.9 (15.0; 28.2)	40.9 (37.4; 44.5)	38.2 (30.9; 46.6)	11.5 (9.9; 13.2)	16.6 (14.6; 18.8)	20.2 (17.5; 23.3)	56.7 (52.5; 60.8)	34.5 (29.7; 39.5)
Peru	49.6 (43.6; 55.5)	21.2 (17.2; 26.0)	62.5 (58.4; 66.5)	16.2 (14.4; 19.5)	19.1 (17.1; 21.3)	15.4 (13.7; 17.2)	2.2 (0.009; 4.9)	71.2 (67.3; 74.9)	28.8 (25.6; 32.3)
Suriname	51.1 (41.3; 60.9)	25.1 (19.9; 31.5)	43.9 (39.5; 48.4)	30.9 (23.0; 40.8)	32.7 (27.9; 37.9)	19.0 (16.6; 21.6)	32.7 (26.7; 39.4)	49.8 (43.6; 56.1)	42.2 (38.9; 45.6)
Uruguay	54.2 (52.4; 56.1)	22.2 (19.4; 25.8)	60.5 (58.6; 62.4)	17.0 (14.1; 20.4)	7.3 (6.2; 8.5)	15.6 (14.1; 17.2)	-	-	-

^a^The time spent in physical education classes was not considered in the total physical activity indicator

**Fig. 1:**
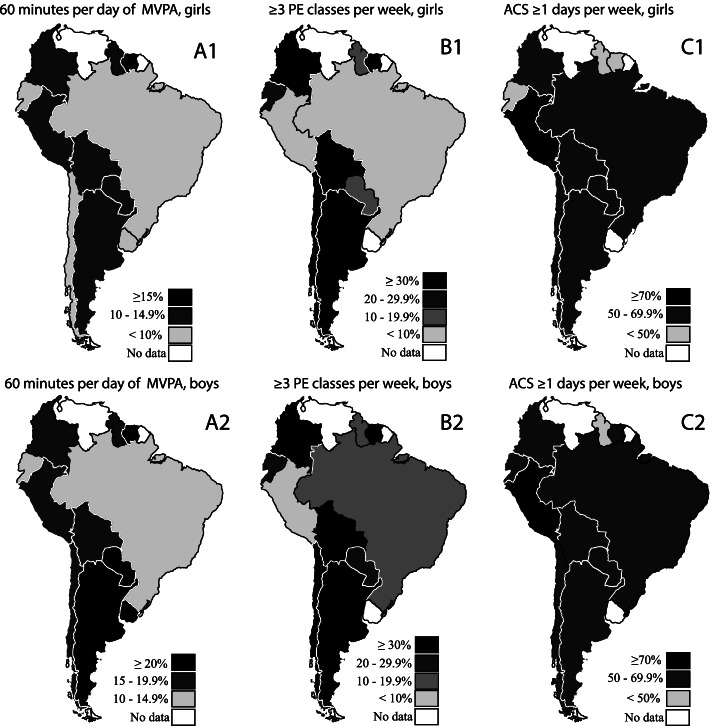
Prevalence of adolescents who accumulate at least 60 min/day of physical activity [girls (A1) and boys (A2)], ≥ 3 Physical Education classes per week [girls (B1) and boys (B2)], and ≥ 1 day/week of active commuting to/from school [girls (C1) and boys (C2)]

Figure 1 presents the prevalence of recommended PA, participation in physical education classes, and active commuting to school, according to gender. Concerning activity domains, the percentage of girls who met PA recommendations was less than 20%. Girls from Suriname had the highest PA indicators, while less than 5% of Brazilian girls met PA recommendations. Among boys, those from Argentina, Paraguay, Suriname, and Uruguay achieved higher PA levels, while Brazilians reported the lowest values. More than 30% of the adolescents from Argentina, Bolivia, Chile, Colombia, and Suriname reported having three or more physical education classes per week. On the other hand, less than 5% of Peruvian adolescents reported three or more physical education classes per week. Less than 50% of the girls from Ecuador, Guyana, and Suriname, and less than 50% of the boys from Guyana, reported active commuting to/from school 1 or more times per week.

**Fig. 2:**
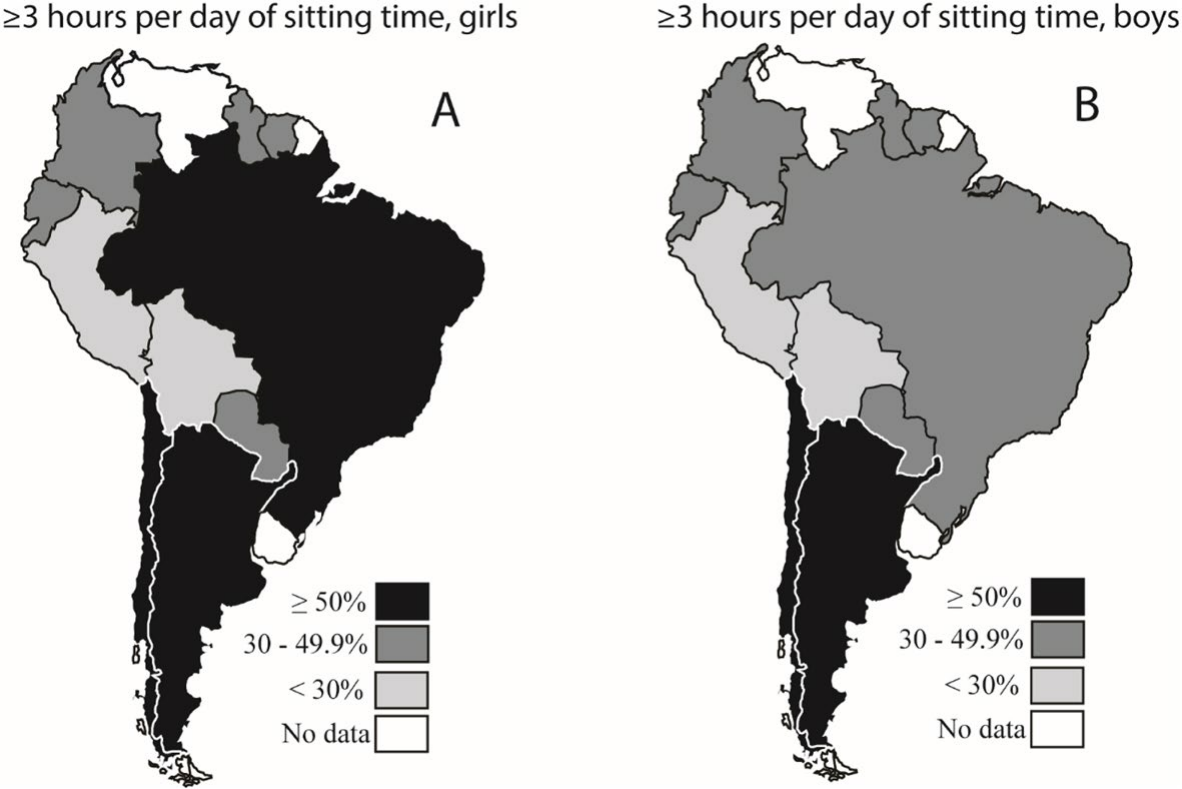
Prevalence of adolescents who spent ≥ 3 h/day sitting outside school [girls (**A**) and boys (**B**)]

Figure 2 shows the prevalence of three or more hours per day of sitting time among adolescents from South America, according to gender. More than 50% of the girls from Argentina, Brazil, and Chile reported three or more sitting hours per day, while the lowest values were seen for Bolivian and Peruvian girls. Among boys, more than 50% of those from Argentina and Chile reported three or more sitting hours per day, while the Bolivian and the Peruvian boys reported the lowest sitting time values. Tables S1 and S2 present the prevalence of outcomes (recommended PA, physical education classes, active commuting to school, and sitting time) according to age group and food security status.

**Fig. 3:**
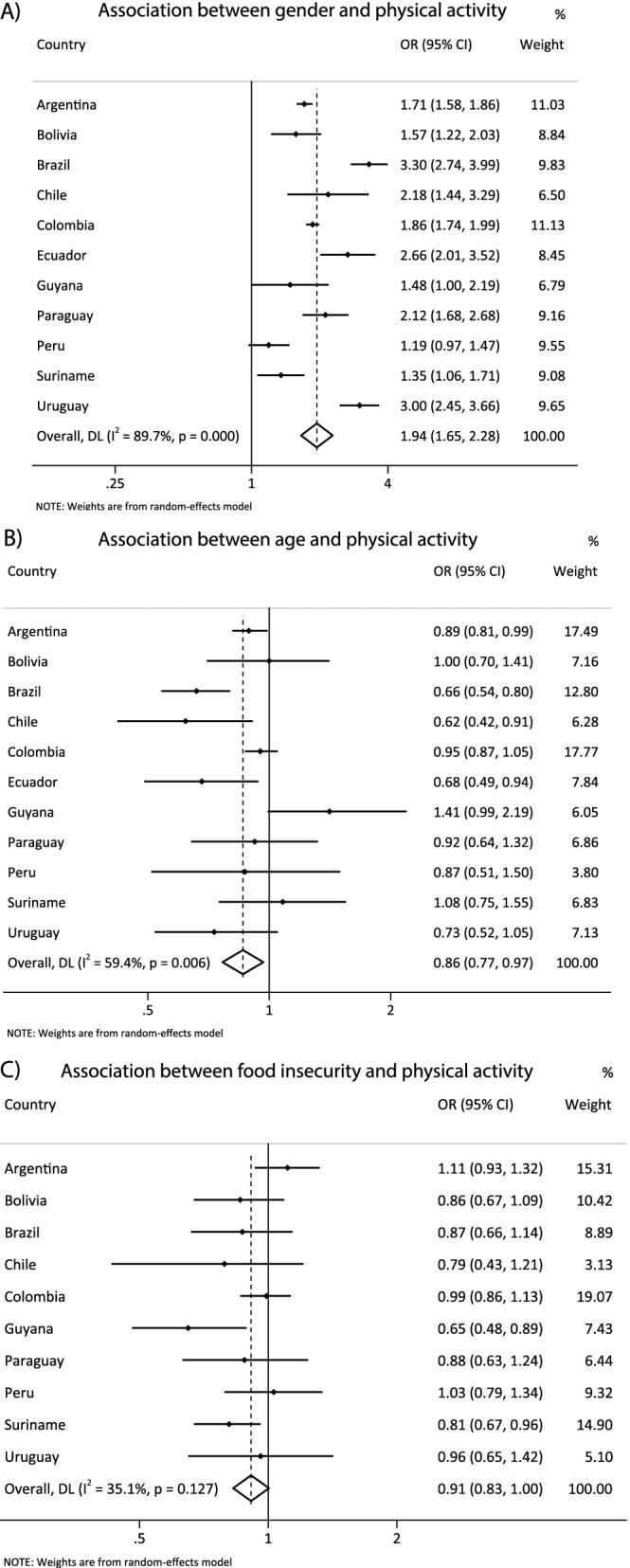
**A** harmonized meta-analysis of the association between sex and physical activity (boys vs girls) with odds ratio results adjusted by age group and food insecurity. **B** harmonized meta-analysis of the association between age and total physical activity (≥ 16 vs 12-13y) with odds ratio results adjusted by gender and food insecurity. **C** harmonized meta-analysis of the association between food insecurity and physical activity (food insecurity vs food security) with odds ratio adjusted by gender and age group. Weights are from the random-effects analysis. OR, odds ratio. 95% CI, 95% confidence interval. Adolescents who met at least 60 min/day of moderate to vigorous physical activity in the previous seven days were classified as active

Figure 3 presents the harmonized meta-analysis of the association between sociodemographic correlates with recommended PA. Compared to girls, boys were more prone to be physically active [OR = 1.94 (CI95% 1.65; 2.28), I^2^ = 89.7%]. Older adolescents had less odds of reach PA guidelines than their youngest pairs [OR = 0.86 (CI95% 0.77; 0.97), I^2^ = 59.4%]. In addition, there was no association between food security status and PA level [OR = 0.91 (CI95%0.83; 1.00], I^2^ = 35.1%].

**Fig. 4:**
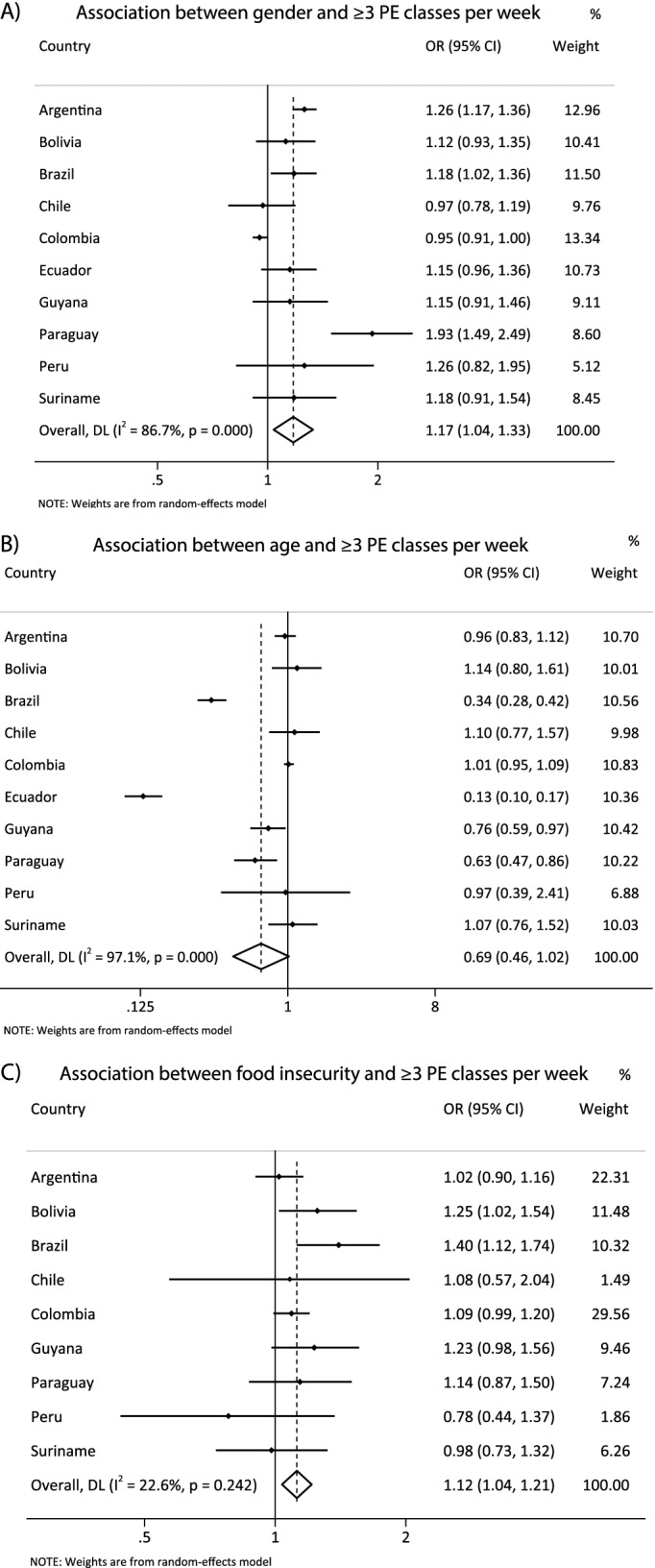
**A** harmonized meta-analysis of the association between sex and physical education classes (boys vs girls) with odds ratio results adjusted by age group and food insecurity. **B** harmonized meta-analysis of the association between age and physical education classes (≥ 16 vs 12-13y) with odds ratio results adjusted by gender and food insecurity. **C** harmonized meta-analysis of the association between food insecurity and physical education classes (food insecurity vs food security) with odds ratio adjusted by gender and age group. Weights are from the random-effects analysis. OR, odds ratio. 95% CI, 95% confidence interval. PE, physical education

Figure 4 presents the harmonized meta-analysis of the association between sociodemographic correlates with participation in physical education classes. Boys were more prone to be engaged in physical education classes, compared to girls [OR = 1.17 (CI95% 1.04; 1.33), I^2^ = 86.7%]. There was no association between age and participation in physical education classes [OR = 0.69 (CI95%0.46; 1.02), I^2^ = 97.1%]. Those who reported food insecurity were more prone to be engaged in physical education classes compared to those who reported food security [OR = 1.12 (CI95%1.04; 1.21), I^2^ = 22.6%].

**Fig. 5:**
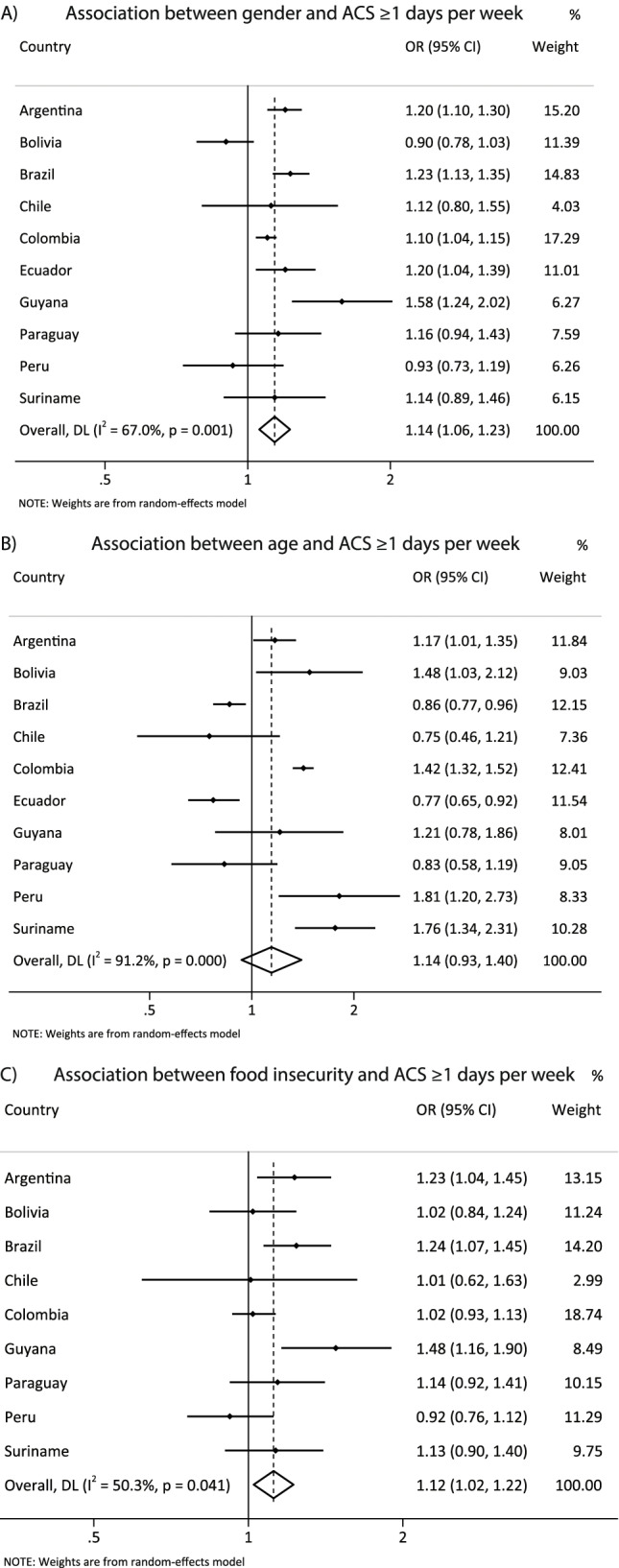
**A** harmonized meta-analysis of the association between sex and active commuting to school (boys vs girls) with odds ratio results adjusted by age group and food insecurity. **B** harmonized meta-analysis of the association between age and active commuting to school (≥ 16 vs 12-13y) with odds ratio results adjusted by gender and food insecurity. **C** harmonized meta-analysis of the association between food insecurity and active commuting to school (food insecurity vs food security) with odds ratio adjusted by gender and age group. Weights are from the random-effects analysis. OR, odds ratio. 95% CI, 95% confidence interval. ACS, active commuting to school

Figure 5 presents the harmonized meta-analysis of the association between sociodemographic correlates with active commuting to school. Boys were more likely to be active during commuting to school compared to girls [OR = 1.14 (CI95%1.06; 1.23), I^2^ = 67.0%]. No association was observed between age and active commuting to school [OR = 1.14 (CI95%0.93; 1.40), I^2^ = 91.2%]. Adolescents with food insecurity had more odds for active commuting to school than their pairs with food security [OR = 1.12 (CI95% 1.02; 1.22), I^2^ = 50.3%].

**Fig. 6:**
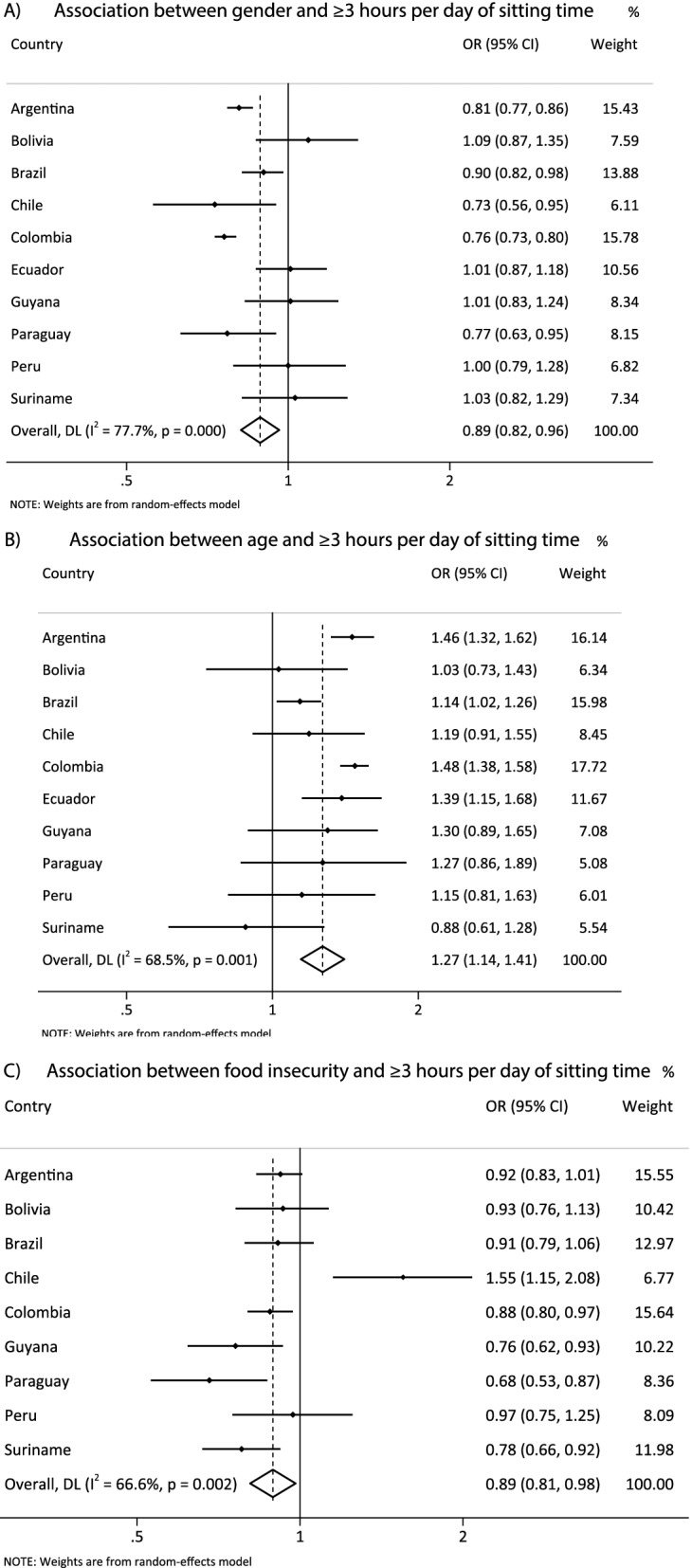
**A** harmonized meta-analysis of the association between sex and sitting time (boys vs girls) with odds ratio results adjusted by age group and food insecurity. **B** harmonized meta-analysis of the association between age and sitting time (≥ 16 vs 12-13y) with odds ratio results adjusted by gender and food insecurity. **C** harmonized meta-analysis of the association between food insecurity and sitting time (food insecurity vs food security) with odds ratio adjusted by gender and age group. Weights are from the random-effects analysis. OR, odds ratio. 95% CI, 95% confidence interval

Figure 6 presents the harmonized meta-analysis of the association between sociodemographic correlates with sitting time. Boys sat less than girls [OR = 0.89 (CI95% 0.82; 0.96), I^2^ = 77.7%], while older adolescents were more likely to spend three or more hours in sitting time than younger adolescents [OR = 1.27 (CI95% 1.14; 1.41), I^2^ = 68.5%]. Adolescents with food insecurity sat less than their pairs with food security [OR = 0.89 (CI95% 0.81; 0.98), I^2^ = 66.6%]. Supplementary table S3 presents the association of intermediary age group categories with the outcomes investigated.

## Discussion

This study aimed to identify the prevalence and sociodemographic correlates of domain-specific PA and sitting time among South American adolescents. The prevalence of active adolescents ranged from 7.5% (Brazil) to 19.0% (Suriname). The percentage of students who reported three or more physical education classes per week was from 2.2% (Peru) to 37.5% (Argentina and Colombia), while active commuting to school ranged from 43.1% (Guyana) to 67.8% (Argentina). In addition, we noted that the prevalence of sitting for three or more hours per day was from 24.6% (Bolivia) to 55.6% (Argentina). Our results revealed that boys were more prone to be physically active, engaged in physical education classes, active in commuting to school, and less likely to present higher sitting time compared to girls. Older adolescents (16y +) were less active and more likely to spend three or more hours in sitting time than younger adolescents (12-13y). Adolescents with food insecurity presented a higher frequency of physical education classes and active commuting to school per week, as well as had less higher sitting time than their food security pairs.

Our results extend previous findings showed by Aguilar-Farias et al. [8] to other countries (Brazil and Paraguay). We also included nationally representative data from Colombia and Ecuador, as well as updating the data from Argentina (from 2012 to 2018) and Suriname (from 2009 to 2016) [8]. Our findings also extended the recent gender inequalities in PA presented by Brazo-Sayavera et al. [10] for other exposures, such as age group and food security status.

We noted heterogeneity in the percentage of adolescents who reported three or more physical education classes. For instance, approximately one third of students from Argentina, Bolivia, Chile, Colombia, and Suriname reported at least three physical education classes per week, while less than 10% of the Brazilians and Peruvians reported three or more physical education classes per week. UNESCO [25] reported that in 2013, approximately 21% of Latin America countries were not implementing physical education classes according to obligations or expectations, which may be linked to several factors, such as the autonomy of schools concerning the weekly frequency of physical education classes, and/or the unavailability of adequate infrastructure in schools [25]. In addition, although it is relevant to recognize that the number of Latin American countries with prescribed national physical education curricula is increasing, the weekly frequency of these classes is still low [8, 25]. These results demonstrate the need for national efforts to promote physical education classes across South American countries.

The prevalence of active commuting to school was from 43.1% (Guyana) to 67.8% (Argentina). Indeed, variations in active commuting to school between countries are expected, and may be related to differences in total population, population density, and gross domestic product [26]. Moreover, countries with a low prevalence of active commuting to school should consider implementing policies (i.e., investments in resources, public health regulations) and changes in the built environment (i.e., bike lanes and walking routes to school) to effectively promote active commuting to school.

We also observed variation in sitting time between countries. While less than 30% of adolescents in Peru and Bolivia reported sitting for three or more hours a day, in Argentina, Brazil, Colombia, and Chile, this was reported by more than 40% of the students. These findings show that strategies aimed at reducing sitting time may have different priorities across countries.

We found that boys were more likely to be physically active, and to participate in physical education classes than girls. These findings corroborate previous studies [27, 28] and highlight a gender inequality related to PA across South American countries. Gender inequality in PA is a worldwide challenge, and previous studies have discussed possible explanations, such as parental influence, social support [15, 29], and stereotypes [30]. In addition, our results also revealed that the magnitude of the associations between gender and PA domains differed across the countries observed. For instance, boys were more physically active than girls in almost all countries, while gender disparities related to physical education classes (Argentina, Brazil, and Paraguay) and active commuting to school (Argentina, Brazil, Colombia, Ecuador, and Guyana) were less frequent, which may be related to the high disparities in leisure-time PA observed previously [16]. Beyond the reduction in gender inequality related to leisure-time PA, the abovementioned countries should seek to reduce gender inequality related to physical education classes, given the beneficial effects of participating in these classes on health [31] and the importance of physical education classes for adolescents to reach the physical activity recommendation [32, 33].

The meta-analysis revealed that older adolescents were less prone to be active than their youngest pairs. Likewise, important results were observed concerning specific countries, where the oldest adolescents from Brazil, Ecuador, Guyana, and Paraguay were less likely to have three or more physical education classes per week than their youngest pairs. Decreasing PA during adolescence has been reported in the literature and different explanations have been suggested, such as lack of time to practice PA and decreasing family support over time [34]. However, our study could raise some insights that may ameliorate this scenario. In this sense, promoting more weekly physical education classes and stimulating the participation of older adolescents, especially from above mentioned countries, in these classes could reduce barriers to PA and increase the odds of reaching the international guidelines.

Older adolescents (16y +) were also more sedentary compared to younger adolescents (12-13y). Our results corroborate findings from high and low-middle- income countries [24, 35], which might be related to increased use of the internet and greater sitting time to perform tasks [35]. Although older adolescents had a higher probability for elevated sitting time (≥ three hours per day), countries such as Argentina, Brazil, Chile, and Colombia presented an elevated prevalence of these behaviors even among the youngest adolescents. Possible explanations for this might be related to high rates of urbanization accompanied by higher access to the internet in these countries [12], which may facilitate access to electronic devices. However, more studies are needed to confirm this hypothesis. Therefore, countries should consider actions to guide the population, such as advising parents and students about the importance of limiting sedentary time [36].

Our results revealed that adolescents with food insecurity reported a higher frequency of weekly physical education classes than their peers in a food security situation. This unexpected association may occur because adolescents with food insecurity could attend schools more often, since schools represent a safe environment against some social problems, such as food insecurity [37]. Especially in low- and middle-income countries, physical education classes can be one of the few free-of-charge opportunities for access to sport and different types of structured PA, which might help to reduce existing social inequalities related to PA [33]. Likewise, adolescents with food insecurity had more odds for active commuting to school, which might the preferable way to reach the school given that walking or cycling represents low costs than other passive transportation modes. On other hand, the meta-analysis revealed that the probability of reaching the PA guidelines might to be lower among adolescents with food insecurity [OR = 0.91 (CI95% 0.83; 1.00), I2 = 35.1%]. Although our data do not allow us to analyze leisure PA, previous studies enable us to suggest that the discrepancy in leisure PA could explain discrepancies in PA related to food security status. For instance, a previous cross-national study comparing 52 countries also found that adolescents in the highest wealth quintile were more active in leisure than their peers in the lowest quintile, which can explain the inequality in PA [16]. Therefore, policies to stimulate PA outside school should also be focused on adolescents with economic disadvantage.

Pooled analysis revealed that adolescents who reported food insecurity were less prone to sit more. These findings corroborate a study carried out with 66 low and middle-income countries [24]. Plausible explanations may be related to the greater access to electronic devices by individuals with higher socioeconomic conditions, as well as the need for food-insecure adolescents to be involved in other activities, such as occupational ones [38]. On the other hand, food insecurity was associated with higher sitting time only in Chile. Chile presents the highest income among the countries analyzed, and interestingly, a review carried out by Mielke et al. [7] revealed that contrary to low-middle income countries, food-insecure adolescents are more prone to present higher sedentary behavior in high-income countries, which highlights the need for country-specific interventions.

### Strengths and limitations

Our results need to be interpreted with caution. Studies on the reliability and validity of the questions used in the present study were not available for the countries analyzed. Thus, it is not possible to estimate whether the differences observed between the countries could be partly explained due to biases in the self-reported estimates. In general lines, a previous review showed that self-reported measures tend to overestimate PA, especially among girls [39]. Therefore, it is possible that the gender differences related to PA could be even greater than our estimates. Also, younger adolescents frequently report less accurate information on PA than their older peers [40], however, the direction of this bias is unknown. Similarly, how the report of PA according to food insecurity is unknown. However, the directions of our findings are in line with studies carried out with objective measures [41], reinforcing that the inequalities that we found in PA and sitting time are not overridden by bias in the self-report.

The strengths of this study are the use of a large sample from 11 South American countries and the use of comparable instruments to assess domains of PA and sitting time as well as to identify the sociodemographic correlates. On the other hand, some limitations need to be pointed out. 1) The surveys were carried out in different years, which may introduce bias in the analysis. 2) The PeNSE, ENSE, and ENSANUT studies adopted different sampling procedures to the GSHS survey, which could lead to potential differences. However, we minimized some of these differences by seeking to harmonize the age groups. 3) There were slight differences in the questionnaires across surveys. 4) PA and sitting time were assessed by self-reported questionnaires, which can lead to memory, cultural and social desirability biases. Also, information on reliability or validity was not available for the countries analyzed. However, given that technological and financial resources are not always feasible to assess objective PA and sedentary behavior data, self-reported questionnaires have been widely used by the scientific community to guide actions in PA surveillance [6, 17]. 5) GSHS, PeNSE, and ENSE are surveys restricted to adolescents who are in school, which limits the extrapolation of results to adolescents not enrolled in schools. Lack of representativeness of adolescents who do not attend school is a challenge in surveillance of PA and sedentary behavior in several countries, and additional efforts to include this population in future studies are needed [42]. 6) It was not possible to estimate the type of sitting time (e.g., TV-viewing, computer use, listening to music), and future studies could further explore this information, given that health outcomes are dependent on the types of sedentary behaviors performed.

## Conclusions

We observed a low prevalence of PA among South American countries, which ranged from 7.5% (Brazil) to 19.0% (Suriname). The prevalence of sitting three or more hours per day presented variations between countries, with values ranging from 24.6% (Bolivia) to 55.6% (Argentina). Thus, strategies aimed at reducing sitting time may have different priorities across countries. In addition, South American boys are more active than girls in different contexts (moderate to vigorous PA, physical education classes, and active commuting to school). Food insecurity is related to higher amounts of weekly physical education classes and active commuting to school. Girls and older adolescents (≥ 16 years old) are the most sedentary groups, which demonstrates the importance of targeting this behavior differently in interventions and policies aimed at reducing sedentary time.

## Supplementary Information

**Additional file 1.** Supplementary Material A.

**Additional file 2.** Chart 1 - Surveys characteristics.

**Additional file 3.** Chart 2- Sociodemographic characteristics of South American countries.

**Additional file 4:** **Table S1.** Prevalence of total physical activity, participation in physical education classes, active commuting to schools, and sitting time, according to age group.

**Additional file 5:** **Table S2.** Prevalence of total physical activity, participation in physical education classes, active commuting to schools, and sitting time, according to food insecurity.

**Additional file 6:** **Table S3.** Harmonized meta-analysis of the association of age (14-15 vs 12-13y) with total physical activity, physical education class, active commuting to school and sitting time.

## Data Availability

Datasets from Argentina, Bolivia, Chile, Guyana, Paraguay, Peru, Suriname, and Uruguay are available in Global School-based Student Health Survey website (https://extranet.who.int/ncdsmicrodata/index.php/catalog/GSHS). Data from Brazil (https://www.ibge.gov.br/estatisticas/todos-os-produtos-estatisticas.html), and Ecuador (https://ensanut.insp.mx/) are available on each governmental website. Data from Colombia are available upon request to the *Ministerio de Salud y Protección Social*. Researchers can apply to use the ENSE (Colombia) resource and access the data used (www.minsalud.gov.co).

## Abbreviations

PA: Physical activity
GSHS: Global School-based Student Health Survey
PeNSE: Brazil National School Health Survey
ENSE: Colombia National School Health Survey
ENSANUT: Ecuador National Health and Nutrition Survey
