# Supplementary material for: Prevalence and sociodemographic correlates of physical activity and sitting time among South American adolescents: a harmonized analysis of nationally representative cross-sectional surveys
Source: Int J Behav Nutr Phys Act. 2022 May 8;19:52. doi: 10.1186/s12966-022-01291-3 (PMC9080195; doi:10.1186/s12966-022-01291-3)
Supplement: Supplementary file 1 — Additional file 1. Supplementary Material A. [file 12966_2022_1291_MOESM1_ESM.docx]

**Supplementary Material A**

**Global School Health Survey (GSHS)**

The GSHS aims to provide data about risk and protective factors among students. The GSHS was developed by the World Health Organization (WHO) and the Centers for Disease Control and Prevention (CDC) in collaboration with UNICEF, UNESCO, and UNAIDS [1]. A 2-stage sample design was used in the GSHS: 1) the schools, selected with probability proportional to enrollment size, reflect the first level; 2) the classrooms, which were randomly selected, and all students in selected classes were eligible to participate. The sample was composed of students from Argentina (n = 56933), Bolivia (n = 3696), Chile (n = 2049), Guyana (n = 2392), Paraguay (n = 3149), Peru (n = 2882), Suriname (n = 2126), and Uruguay (n = 3524). Considering the exclusion by age and missing data, the final sample using GSHS was composed of students from Argentina (n = 52423), Bolivia (n = 3241), Chile (n = 1878), Guyana (n = 2193), Paraguay (n = 2848), Peru (n = 2793), Suriname (n = 2004), and Uruguay (n = 3393).

**Brazil National School Health Survey (Pesquisa Nacional de Saúde do Escolar - PeNSE)**

The PeNSE seeks to assess risk factors for health among students enrolled in Brazilian public and private schools. Briefly, the data were collected by the Brazilian Institute of Geography and Statistics, Ministry of Health, and Ministry of Education [2]. The 2015 PeNSE adopted a cluster sampling procedure that included schools in the 26 state capitals and Federal District and 26 other municipalities, resulting in 53 strata. In capitals, the schools composed the first level, while classrooms were the second level. In non-capitals, three stages were used, with municipalities as primary, schools as second, and classrooms as the third level. The PeNSE is composed of two databases (Sample 1 and Sample 2). We used Sample 2 due to the greater representativeness of students aged 13-17y. The sample was composed of 16,556 subjects. Considering the exclusion by age and missing data, the final sample used by the present study was 14,321.

**Colombia National School Health Survey (*Encuesta Nacional de Salud Escolar* – ENSE)**

The ENSE aims to assess factors related to the health of students aged 13-17 years, such as food behavior, physical activity, alcohol use, and oral health [3]. The 2017 ENSE adopted a cluster sampling in 3 stages, with municipalities as primary, schools as second, and classrooms as the third level. The sample was composed of 79,640 subjects. Considering the exclusion by age and missing data, the final sample used by the present study was 72,808.

**Ecuador National Health and Nutrition Survey (*Encuesta Nacional de Salud y Nutrición* - ENSANUT)**

The 2018 ENSANUT sought to raise information on risk and protective factors for health among Ecuadorian population [4]. The sample was obtained by three-stage sampling. The first stage was formed by census tracts; the second by household; and the third by residents. In each selected household (randomly), a child or adolescent 5-17 years old was selected using the nearest birthday method. The sample was composed of 23,621 subjects. Considering the exclusion by age and missing data, the final sample used by the present study was 8,999 adolescents.

**References**

1. World Health Organization. Global School-Based Student Health Survey [Internet]. [cited 2021 Jun 8]. Available from: https://www.who.int/teams/noncommunicable-diseases/surveillance/systems-tools/global-school-based-student-health-survey

2. Instituto Brasileiro de Geografia e Estatística (IBGE). Pesquisa Nacional de Saúde do Escolar, (PeNSE), 2015. Rio de Janeiro: IBGE; 2016.

3. Ministerio de Salud y Protección Social. Encuesta Nacional de Salud en Escolares (ENSE). 2020;

4. Instituto Nacional de Estadítica y Censos. Encuesta Nacional de Salud y Nutrición. Quito; 2019.
