# Supplementary material for: Prevalence and sociodemographic correlates of physical activity and sitting time among South American adolescents: a harmonized analysis of nationally representative cross-sectional surveys
Source: Int J Behav Nutr Phys Act. 2022 May 8;19:52. doi: 10.1186/s12966-022-01291-3 (PMC9080195; doi:10.1186/s12966-022-01291-3)
Supplement: Supplementary file 2 — Additional file 2. Chart 1 - Surveys characteristics. [file 12966_2022_1291_MOESM2_ESM.docx]

**Chart 1 - Surveys characteristics.**

| Country | Survey year | Sample size | Sample (≥ 12 years old) | Missing* | Final sample |
| --- | --- | --- | --- | --- | --- |
| Argentina | 2018 | 56933 | 56933 | 4510 | 52423 |
| Bolivia | 2012 | 3696 | 3649 | 408 | 3241 |
| Brazil | 2015 | 16556 | 14484 | 163 | 14321 |
| Chile | 2013 | 2049 | 2045 | 167 | 1878 |
| Colombia | 2017 | 79640 | 79640 | 6832 | 72808 |
| Ecuador | 2018 | 23621 | 9891 | 892 | 8999 |
| Guyana | 2010 | 2392 | 2373 | 180 | 2193 |
| Paraguay | 2017 | 3149 | 3126 | 278 | 2848 |
| Peru | 2010 | 2882 | 2868 | 75 | 2793 |
| Suriname | 2016 | 2126 | 2116 | 112 | 2004 |
| Uruguay | 2012 | 3524 | 3510 | 117 | 3393 |

Note: *considering the sample with participants aged ≥12 years.
