# Supplementary material for: Prevalence and sociodemographic correlates of physical activity and sitting time among South American adolescents: a harmonized analysis of nationally representative cross-sectional surveys
Source: Int J Behav Nutr Phys Act. 2022 May 8;19:52. doi: 10.1186/s12966-022-01291-3 (PMC9080195; doi:10.1186/s12966-022-01291-3)
Supplement: Supplementary file 3 — Additional file 3. Chart 2- Sociodemographic characteristics of South American countries. [file 12966_2022_1291_MOESM3_ESM.docx]

**Chart 2- Sociodemographic characteristics of South American countries.**

|  | Urban Population (%) | Life expectancy at birth (total, years) | Individuals using the internet (per 100 inhabitants) | GINI |
| --- | --- | --- | --- | --- |
| Argentina (2018) | 91.9 | 76.5 | 74.2^d^ | 41.3 |
| Bolivia (2012) | 67.3 | 69.9 | 35.3 | 46.6 |
| Brazil (2015) | 85.7 | 74.9 | 58.3 | 51.9 |
| Chile (2013) | 82.2 | 79.3 | 58 | 45.8 |
| Colombia (2017) | 80.4 | 76.9 | 62.2 | 49.7 |
| Ecuador (2018) | 63.8 | 76.8 | 54.1^c^ | 45.4 |
| Guyana (2010) | 26.6 | 68.0 | 29.9 | 45.1^a^ |
| Paraguay (2017) | 59.2 | 73.9 | 61.0 | 48.5 |
| Peru (2010) | 76.4 | 74.4 | 34.7 | 41.5 |
| Suriname (2016) | 66.0 | 71.3 | 45.4 | 57.9^b^ |
| Uruguay (2012) | 59.9 | 77.0 | 54.4 | 39.9 |

Note: Data from 1998^a^, 1999^b^, 2016^c^, 2017^d^. https://data.worldbank.org/.
