# Supplementary material for: Prevalence and sociodemographic correlates of physical activity and sitting time among South American adolescents: a harmonized analysis of nationally representative cross-sectional surveys
Source: Int J Behav Nutr Phys Act. 2022 May 8;19:52. doi: 10.1186/s12966-022-01291-3 (PMC9080195; doi:10.1186/s12966-022-01291-3)
Supplement: Supplementary file 4 — Additional file 4: Table S1. Prevalence of total physical activity, participation in physical education classes, active commuting to schools, and sitting time, according to age group. [file 12966_2022_1291_MOESM4_ESM.docx]

**Table S1 - Prevalence of total physical activity, participation in physical education classes, active commuting to schools, and sitting time, according to age group.**

|  | Total physical activity | | | Physical education | | | Active commuting | | | Sitting time | | |
| --- | --- | --- | --- | --- | --- | --- | --- | --- | --- | --- | --- | --- |
|  | 12 to 13 | 14 to 15 | 16+ | 12 to 13 | 14 to 15 | 16+ | 12 to 13 | 14 to 15 | 16+ | 12 to 13 | 14 to 15 | 16+ |
| Argentina | 17.0 (15.9; 18.3) | 16.2 (15.2; 17.3) | 15.7 (14.7; 16.8) | 37.3 (34.9; 39.8) | 38.2 (36.2; 40.1) | 36.6 (34.4; 38.8) | 65.2 (62.2; 68.1) | 68.3 (65.4; 71.0) | 68.8 (65.9; 71.5) | 49.6 (47.1; 52.2) | 55.9 (53.7; 58.2) | 58.9 (57.0; 60.7) |
| Bolivia | 14.2 (11.3; 17.7) | 14.0 (12.0; 16.3) | 14.4 (11.7; 17.7) | 32.5 (26.6; 39.0) | 29.5 (26.4; 32.8) | 35.6 (31.1; 40.3) | 59.3 (53.3; 65.0) | 65.4 (60.3; 70.3) | 68.1 (61.6; 74.0) | 23.4 (18.7; 28.8) | 25.3 (22.3; 28.6) | 23.9 (20.2; 28.0) |
| Brazil | 8.8 (7.8; 9.8) | 8.8 (7.0; 9.2) | 6.2 (5.4; 7.1) | 15.4 (14.2; 16.7) | 9.3 (8.2; 10.4) | 5.9 (5.1; 6.9) | 59.4 (57.7; 61.1) | 59.5 (57.5; 61.4) | 55.9 (54.0; 57.9) | 47.2 (45.5; 49.0) | 53.7 (51.7; 55.7) | 50.4 (48.4; 52.3) |
| Chile | 17.1 (13.9; 21.0) | 14.3 (11.4; 17.8) | 11.4 (8.9; 14.3) | 30.7 (27.0; 34.7) | 34.4 (29.2; 40.0) | 32.9 (28.0; 38.2) | 64.4 (58.8; 69.7) | 67.4 (61.9; 72.5) | 57.4 (47.0; 67.2) | 51.11 (45.9; 56.3) | 53.8 (48.0; 59.4) | 55.8 (49.6; 61.7) |
| Colombia | 15.3 (14.3; 16.3) | 15.1 (14.5; 15.8) | 14.8 (14.1; 15.5) | 37.3 (35.9; 38.6) | 37.5 (36.6; 38.4) | 37.6 (36.7; 38.6) | 57.3 (55.9; 58.7) | 62.7 (61.8; 63.6) | 65.6 (64.7; 66.5) | 40.4 (39.0; 41.8) | 46.7 (45.8; 47.6) | 49.8 (48.9; 50.8) |
| Ecuador | 10.7 (0.8; 13.0)* | 10.4 (8.6; 12.6)* | 7.7 (6.2; 9.6)* | 43.2 (40.3; 46.2) | 26.4 (23.7; 29.3) | 8.9 (7.1; 11.0) | 52.6 (49.7; 55.6) | 51.4 (48.3; 54.40 | 46.2 (43.0; 49.4) | 31.6 (28.8; 34.6) | 36.5 (33.6; 39.6) | 39.2 (36.0; 42.4) |
| Guyana | 13.8 (8.1; 22.8) | 15.7 (12.9; 18.9) | 19.1 (15.2; 23.6) | 22.3 (18.3; 27.0) | 17.8 (14.1; 22.1) | 18.0 (15.0; 21.5) | 40.2 (31.6; 49.4) | 43.7 (36.7; 51.0) | 45.4 (38.0; 52.9) | 32.4 (25.1; 40.6) | 36.8 (31.3; 42.7) | 38.6 (33.6; 43.8) |
| Paraguay | 16.6 (13.2; 20.6) | 17.1 (14.1; 20.5) | 16.1 (13.5; 19.1) | 24.6 (20.5; 29.2) | 20.4 (16.8; 24.4) | 17.7 (15.1; 20.7) | 58.7 (50.0; 66.9) | 57.9 (52.7; 62.9) | 54.3 (50.2; 58.4) | 30.9 (25.1; 37.3) | 35.0 (29.3; 41.1) | 35.9 (29.9; 42.3) |
| Peru | 18.2 (13.6; 23.8) | 14.2 (12.6; 15.9) | 16.2 (12.4; 21.0) | 1.7 (0.7; 4.1) | 2.5 (1.0; 6.2) | 1.7 (0.8; 3.6) | 65.2 (58.2; 71.5) | 71.8 (66.9; 76.2) | 77.1 (71.1; 82.2) | 25.9 (21.3; 31.1) | 29.9 (26.2; 33.9) | 28.7 (23.4; 34.6) |
| Suriname | 18.3 (15.5; 21.5) | 19.1 (15.3; 23.5) | 19.4 (15.4; 24.1) | 31.3 (24.9; 38.4) | 33.4 (25.2; 42.7) | 33.0 (26.0; 40.8) | 42.3 (34.2; 50.7) | 49.3 (43.1; 55.5) | 56.8 (50.4; 62.9) | 40.7 (35.0; 46.8) | 46.6 (42.6; 50.5) | 37.3 (31.6; 43.3) |
| Uruguay | 15.5 (13.6; 19.9) | 16.1 (14.2; 18.2) | 12.5 (10.2; 15.3) | - | - | - | - | - | - | - | - | - |
