# Supplementary material for: Prevalence and sociodemographic correlates of physical activity and sitting time among South American adolescents: a harmonized analysis of nationally representative cross-sectional surveys
Source: Int J Behav Nutr Phys Act. 2022 May 8;19:52. doi: 10.1186/s12966-022-01291-3 (PMC9080195; doi:10.1186/s12966-022-01291-3)
Supplement: Supplementary file 5 — Additional file 5: Table S2. Prevalence of total physical activity, participation in physical education classes, active commuting to schools, and sitting time, according to food insecurity. [file 12966_2022_1291_MOESM5_ESM.docx]

**Table S2 - Prevalence of total physical activity, participation in physical education classes, active commuting to schools, and sitting time, according to food insecurity.**

|  | Total physical activity | | Physical education | | Active commuting | | Sedentary bahavior | |
| --- | --- | --- | --- | --- | --- | --- | --- | --- |
|  | Food security | Food insecurity | Food security | Food insecurity | Food security | Food insecurity | Food security | Food insecurity |
| Argentina | 16.1 (15.2; 17.0) | 17.3 (15.4; 19.4) | 37.4 (35.9; 39.0) | 37.9 (34.7; 41.2) | 67.3 (64.8; 69.8) | 71.7 (67.7; 75.3) | 55.8 (54.2; 57.4) | 54.0 (50.6; 57.3) |
| Bolivia | 14.6 (12.7; 16.8) | 12.9 (10.6; 15.6) | 30.0 (27.4; 32.8) | 35.1 (31.8; 38.7) | 64.5 (59.5; 69.1) | 65.0 (61.7; 68.2) | 25.0 (22.0; 28.2) | 23.6 (20.1; 27.4) |
| Brazil | 7.6 (7.0; 8.2) | 6.6 (5.2; 8.4) | 9.2 (8.6; 9.9) | 12.3 (10.3; 14.6) | 57.4 (56.2; 58.6) | 62.5 (59.2; 65.7) | 50.8 (49.6; 52.0) | 48.5 (45.0; 52.0) |
| Chile | 13.9 (12.1; 16.0) | 10.6 (6.5; 16.6) | 32.8 (30.1; 35.6) | 34.5 (22.8; 48.6) | 62.4 (56.8; 67.7) | 62.4 (52.0; 71.7) | 53.2 (49.7; 56.7) | 63.6 (55.8; 70.8) |
| Colombia | 15.0 (14.6; 15.5) | 14.8 (13.2; 16.5) | 37.3 (36.8; 37.9) | 39.4 (37.2; 41.6) | 62.6 (62.0; 63.2) | 63.4 (61.2; 65.6) | 46.7 (46.1; 47.3) | 44.0 (41.8; 46.3) |
| Ecuador | - | - | - | - | - | - | - | - |
| Guyana | 17.5 (14.0; 21.6) | 12.2 (9.6; 15.4) | 17.8 (14.5; 21.6) | 21.2 (18.3; 24.4) | 39.9 (33.0; 47.2) | 49.5 (44.0; 55.1) | 38.1 (33.7; 42.8) | 31.7 (26.6; 37.4) |
| Paraguay | 16.8 (14.9; 18.8) | 15.4 (11.1; 20.9) | 20.0 (17.2; 23.1) | 22.4 (17.4; 28.3) | 56.3 (52.1; 60.5) | 59.6 (53.5; 65.4) | 35.4 30.6; 40.5) | 27.1 (21.4; 33.7) |
| Peru | 15.3 (13.6; 17.1) | 15.6 (12.4; 19.5) | 2.4 (0.9; 5.4) | 1.8 (0.9; 3.6) | 71.5 (67.7; 75.1) | 70.1 (64.4; 75.2) | 29.0 (25.6; 32.6) | 28.2 (23.2; 33.9) |
| Suriname | 20.1 (17.3; 23.2) | 16.6 (14.6; 18.8) | 32.9 (26.5; 40.0) | 32.3 (25.3; 40.3) | 48.6 (42.1; 55.3) | 52.3 (45.6; 58.8) | 44.3 (40.6; 48.1) | 38.0 (34.7; 41.4) |
| Uruguay | 15.6 (14.0; 17.3) | 15.6 (11.8; 20.3) | - | - | - | - | - | - |
