# Supplementary material for: Prevalence and sociodemographic correlates of physical activity and sitting time among South American adolescents: a harmonized analysis of nationally representative cross-sectional surveys
Source: Int J Behav Nutr Phys Act. 2022 May 8;19:52. doi: 10.1186/s12966-022-01291-3 (PMC9080195; doi:10.1186/s12966-022-01291-3)
Supplement: Supplementary file 6 — Additional file 6: Table S3. Harmonized meta-analysis of the association of age (14-15 vs 12-13y) with total physical activity, physical education class, active commuting to school and sitting time. [file 12966_2022_1291_MOESM6_ESM.docx]

**Table S3 - Harmonized meta-analysis of the association of age (14-15 vs 12-13y) with total physical activity, physical education class, active commuting to school and sitting time.**

|  | Total physical activity | | | Physical education classes | | | Active commuting to school | | | Sitting time | | |
| --- | --- | --- | --- | --- | --- | --- | --- | --- | --- | --- | --- | --- |
|  | OR (95% CI) | % weight | I^2^ | OR (95% CI) | % weight | I^2^ | OR (95% CI) | % weight | I^2^ | OR (95% CI) | % weight | I^2^ |
| Argentina | 0.93 (0.83; 1.05) | 23.96 |  | 1.03 (0.93; 1.15) | 12.19 |  | 1.14 (1.00; 1.31) | 14.18 |  | 1.30 (1.17; 1.43) | 22.22 |  |
| Bolivia | 0.98 (0.73; 1.34) | 3.59 |  | 0.87 (0.61; 1.24) | 9.12 |  | 1.30 (0.95; 1.79) | 5.76 |  | 1.11 (0.84; 1.47) | 2.91 |  |
| Brazil | 0.89 (0.73; 1.08) | 8.64 |  | 0.56 (0.48; 0.66) | 11.7 |  | 1.00 (0.90; 1.11) | 16.27 |  | 1.30 (1.02; 1.45) | 7.37 |  |
| Chile | 0.82 (0.59; 1.14) | 3.05 |  | 1.18 (0.90; 1.56) | 10.25 |  | 1.14 (0.87; 1.50) | 7.1 |  | 1.10 (0.85; 1.43) | 3.37 |  |
| Colombia | 0.99 (0.90; 1.08) | 39.85 |  | 1.01 (0.94; 1.08) | 12.43 |  | 1.25 (1.17; 1.34) | 18.72 |  | 1.30 (1.21; 1.39) | 47.41 |  |
| Ecuador | 0.96 (0.71; 1.30) | 3.62 |  | 0.47 (0.39. 0.57) | 11.36 |  | 0.95 (0.80; 1.12) | 12.02 |  | 1.24 (1.03; 1.50) | 6.45 |  |
| Guyana | 1.10 (0.72; 1.39) | 1.4 |  | 0.74 (0.53; 1.04) | 9.37 |  | 1.12 (0.83; 1.52) | 6.15 |  | 1.21 (0.89; 1.65) | 2.39 |  |
| Paraguay | 1.00 (0.72; 1.39) | 3.06 |  | 0.76 (0.59; 0.99) | 10.47 |  | 0.96 (0.71; 1.31) | 6.05 |  | 1.22 (0.88; 1.67) | 2.22 |  |
| Peru | 0.75 (0.54; 1.03) | 3.18 |  | 1.46 (0.52; 4.09) | 2.98 |  | 1.36 (0.98; 1.88) | 5.53 |  | 1.22 (0.98; 1.53) | 4.6 |  |
| Suriname | 1.07 (0.80; 1.43) | 3.93 |  | 1.11 (0.84; 1.48) | 10.13 |  | 1.33 (1.04; 1.69) | 8.22 |  | 1.28 (0.95; 1.73) | 2.54 |  |
| Uruguay | 0.97 (0.76; 1.23) | 5.72 |  | - |  |  |  |  |  |  |  |  |
| **Overall** | 0.95 (0.90; 1.01) | 100 | 0.00% | 0.84 (0.68; 1.02) | 100 | 91.50% | 1.13 (1.03; 1.24) | 100 | 67.40% | 1.28 (1.22; 1.34) | 100 | 0.00% |

Note: Odds Ratio results adjusted by gender and food insecurity. Weights are from the random-effects analysis. OR, odds ratio. 95% CI, 95% confidence interval.
